# *In silico* Comparison of Test-and-Cull Protocols for Bovine Tuberculosis Control in France

**DOI:** 10.3389/fvets.2018.00265

**Published:** 2018-10-23

**Authors:** Héléna Ladreyt, Mathilde Saccareau, Aurélie Courcoul, Benoit Durand

**Affiliations:** ^1^French School of Veterinary Services (ENSV), Lyon, France; ^2^Mathematical Modelling of Infectious Diseases Unit, Institut Pasteur, Paris, France; ^3^Epidemiology Unit, Paris-Est University, Laboratory for Animal Health, French Agency for Food, Environment and Occupational Health and Safety (ANSES), Maisons-Alfort, France

**Keywords:** bovine tuberculosis, dynamic modeling, cattle herd, disease control, test-and-cull protocol

## Abstract

Whole depopulation of cattle herds (WHD) confirmed infected by bovine tuberculosis (bTB) has led since the 1950s to a drop of herd incidence in France below 0.1% in 2000, justifying the current officially bTB free (OTF) status of the country. However, this protocol is expensive, ethically questionable, and difficult for breeders to accept because the number of confirmed animals in an infected herd is often very low. A test-and-cull protocol combining at least three screening sessions of the entire herd followed by the slaughter of all the non-negative animals has been used for some years. The aim of this work was to evaluate *in silico* the epidemiological effectiveness, the public costs and the acceptability to farmers of this test-and-cull protocol as well as of several ones. A stochastic compartmental model of within-herd bTB spread was used. Six test-and-cull protocols were compared: two versions of the official protocol and four alternatives with varying delays between screenings, and varying tests used. Protocols were simulated for an average French beef herd, and compared to WHD. Three key indicators were computed: the failure probability of the protocol (a failure being defined as an herd recovering its OTF status recovery while still infected, indicator of epidemiological effectiveness), its overall public cost and the percentage of farmers who would have dropped it to switch to WHD (indicator of acceptability to farmers). Failure probability ranged from 1.4 to 12.4% and was null (by definition) for WHD. The median cost varied between 2.7 and 78 K€ for the test-and-cull protocols, vs. 120 K€ for WHD. The percentage of dropout ranged from 7.8 to 22%. The optimal tradeoff between epidemiological effectiveness, public costs, and acceptability to farmers was obtained for protocols with an increased delay (6 months instead of 2 in the currently used protocol) between the last two screening sessions, with either 3 or 2 screening sessions. This study may help improving the official test-and-cull protocol applied in France under European Union regulation, by suggesting alternative protocols, very effective, cheaper, and more acceptable than WHD.

## Introduction

The European Commission recognizes most of the European countries officially bTB free (OTF) but the infection remains endemic in cattle herds in several parts of Europe such as Spain, Ireland, some regions of United Kingdom, and some regions of Italy (1–5). France has been OTF since 2001 (Decision 2001/26/EC), but this status, which is essential for trade, is threatened by the upsurge of the disease in cattle farms since 2004 (6).The surveillance and control of bTB in Europe focus on animal screening (in slaughterhouses and farms) and the elimination of infection in detected infected herds. The surveillance and control protocols vary according to the local epidemiological situation. In France, in areas with recent outbreaks or where wildlife is involved in bTB transmission, surveillance consists in yearly screening tests, and disease control protocols tend to be drastic. In areas where *Mycobacterium bovis* has not been detected for a long time, surveillance can be reduced to animal screening every 4 years, or can only be based on meat inspection in slaughterhouses, although screening tests are still performed each year in specific herds considered at-risk (e.g., herds producing raw milk or those identified by contact-tracing from recent outbreaks). Disease elimination protocols implemented in outbreaks have also evolved in France since the very first mandatory measures defined in the 1950s. Protocols became progressively more drastic until 1999, when whole depopulation became mandatory in herds where the infection by *M. bovis* had been confirmed (Ministerial Decree of 4 May 1999). These measures allowed an almost complete eradication of bTB in France, reducing the prevalence of 25% of herds infected in 1955 to less than 0.1% in 2001, thus justifying obtaining the officially bTB free (OTF) status (6, 7).

In this context, whole depopulation remains the recommended method of disease elimination in infected herds. In practice, however, it becomes less and less adapted to the epidemiological situation in France, where bTB prevalence remains very low (around 100 herds reported as infected per year) despite a slight increase since 2004 (6). This measure first entails significant public costs, partly due to compensations paid to farmers for all the slaughtered animals, of which only a small number are infected: the average cost for an outbreak (compensations and disinfection) was 107 k€ in 2014 (6). More globally, in 2012, of the 193 million euros dedicated to surveillance and eradication plans by the EU, more than a third was attributed to tuberculosis (8), and more than 17 million euros were spent for bTB control in 2014 in France (6). Studies are then needed to reduce bTB control costs as it is done in other countries (9). A second drawback of whole herd depopulation is that this disease elimination protocol is difficult to accept for obvious ethical reasons for farmers and welfare reasons for animals. In addition, since the within-herd prevalence of the infection is low, very few of the slaughtered animals are confirmed to be infected. Although this does not imply that negative animals are uninfected, the breeders have the impression to have unnecessarily culled their animals. This low acceptability of the protocol can lead to a real lack of effectiveness of the control strategy, which is a source of concern for the animal health authorities (10). Finally, besides cost and acceptability problems, the effectiveness of whole herd depopulation can sometimes be questioned with the recurrence of bTB in some farms after restocking (11). These factors motivated the evolution of French regulations toward a gradual reintroduction of a test-and-cull protocol. This selective slaughter of only animals reacting to a combination of tests was thus authorized under certain conditions throughout France in 2014.

Only one test-and-cull protocol is currently authorized, which has not been evaluated yet. The question arises whether it may be improved in terms of epidemiological effectiveness, public costs and acceptability to the farmer. The current test-and-cull protocol provides for three screening tests with 2 months between each. All three controls have to be consecutively negative to allow the herd regaining its OTF status. The two first screening tests associate single intradermal tuberculin skin test (SIT), gamma-interferon assay (IFN-γ), and a serology test, whereas the third one uses the single intradermal comparative cervical skin test (SICCT). In practice, in areas where the infection by atypical mycobacteria is known to be frequent in cattle, SIT may be replaced by SICCT to increase the specificity of screenings.

The aim of this work was to compare, through modeling and simulation, several test-and-cull scenarios in a bTB-infected farm, to determine the most epidemiologically effective scenario, while evaluating its public costs and its acceptability to the farmer.

## Materials and methods

### Model

The model was a stochastic compartmental model operating in monthly time steps. Only females involved in reproduction were represented, as other animals were assumed to play only a minor role in the epidemiological system, either because of their short lifespan (calves, beef cattle), or because they are very few (bulls). Each heifer or cow was represented by its age (in years) and its health state, with S (susceptible) for non-infected animals, E (latent) for infected animals that do not excrete the bacteria yet and do not have lesions, and I (infectious) for infected animals having lesions and excreting the bacteria. The dynamics of bTB in a cattle herd, from the *M. bovis* introduction to the elimination of infection was represented by three processes:

- The demographic process: The age structure resulted from the cull of animals, the culling rate being assumed to vary according to the month and to the age class (null for heifers, >0 for cows). The size of the herd was assumed constant and the herd closed: slaughtered animals (because of routine slaughter or due to disease control) were replaced by young animals born in the same herd.
- The infectious process: animals were assumed to be grouped into batches according to their age class, distinct batches being kept in separate buildings or on distant pastures. The transmission of *M. bovis* was thus assumed to only occur between animals of the same batch. However, because of the aging of animals, the composition of batches changed every year, and animal transfers between batches allowed *M. bovis* to spread inside the herd. *M. bovis* transmission intensity was assumed to vary according to whether the animals are housed inside a stable (high intensity of within-batch transmission) or allowed to graze (low intensity of within-batch transmission), the transmission parameter was thus assumed to vary accordingly.
- The detection and control process: it combined ante mortem tests (SIT, SICCT, IFN-γ, and serology), post mortem and confirmation tests (routine or detailed carcass inspection, PCR, bacterial culture, and histology), as well as the culling of all (whole herd depopulation) or specific (test-and-cull) animals. At the individual level, it was assumed that ante-mortem tests may allow detecting animals in the E and I states (according to the sensitivities of these tests), whereas only animals in the I state could be detected by carcass inspection (again according to the sensitivity of routine or detailed inspection). The detection and control process was represented by a succession of steps, one or several tests being implemented at each step on one or several animals. Moves from one step to another depended on the results of these tests, and on those of routine carcass inspection. This representation allowed the model to simulate detection and control programs of arbitrary degree of complexity.

The model parameters were estimated from field data using Approximate Bayesian Computation (ABC) methods. The duration of the latent state was thus estimated 3.5 months (95% credible interval: 2–8 months), the transmission parameter was estimated 0.43 month^−1^ (95% CI: 0.16–0.84) inside buildings, and 0.08 month^−1^ (95% CI: 0.01–0.32) on pastures. Based on these estimates, the model was then validated using an independent dataset.

The model was implemented using the R software. A detailed description of its structure, parameterization and validation can be found in Bekara et al. (12).

### Parameters

#### Demographic and infection process

As most of herds detected infected in France are beef herds, only this type of herd (i.e., breeding and suckling herds) was addressed in our study, and the values of the parameters of the demographic process (Table 1) were chosen to represent a typical French beef herd, in 2017. Three animal batches were considered, 1-, 2-, and 3-year old heifers, and cows with their calves.

**Table 1:** Parameters of the demographic process included in the model of within-herd bTB dynamics.

**Parameter**	Value^*^	**Source**
Size of the herd	141 animals	(13–15)
Maximal age of cows	15 years	(12)
Stabling period	November to March	(12)
Yearly culling rate	35%	(16)
Age of culled animals	≥ 4 years	(12)
Culling period	January to March	(12)

* *Values characterizing of an average French beef cattle herd*.

At the beginning of each simulation, the values of the two transmission parameters (inside buildings and on pastures) and of the duration of the latent period were randomly drawn from the joint posterior distribution produced by the ABC estimation procedure (12). Moreover, the infection was assumed to be brought in the herd by the introduction of a single infected animal (I health state), at a randomly chosen month of the first simulated year.

#### Modeled surveillance and control measures

**Figure 1:**
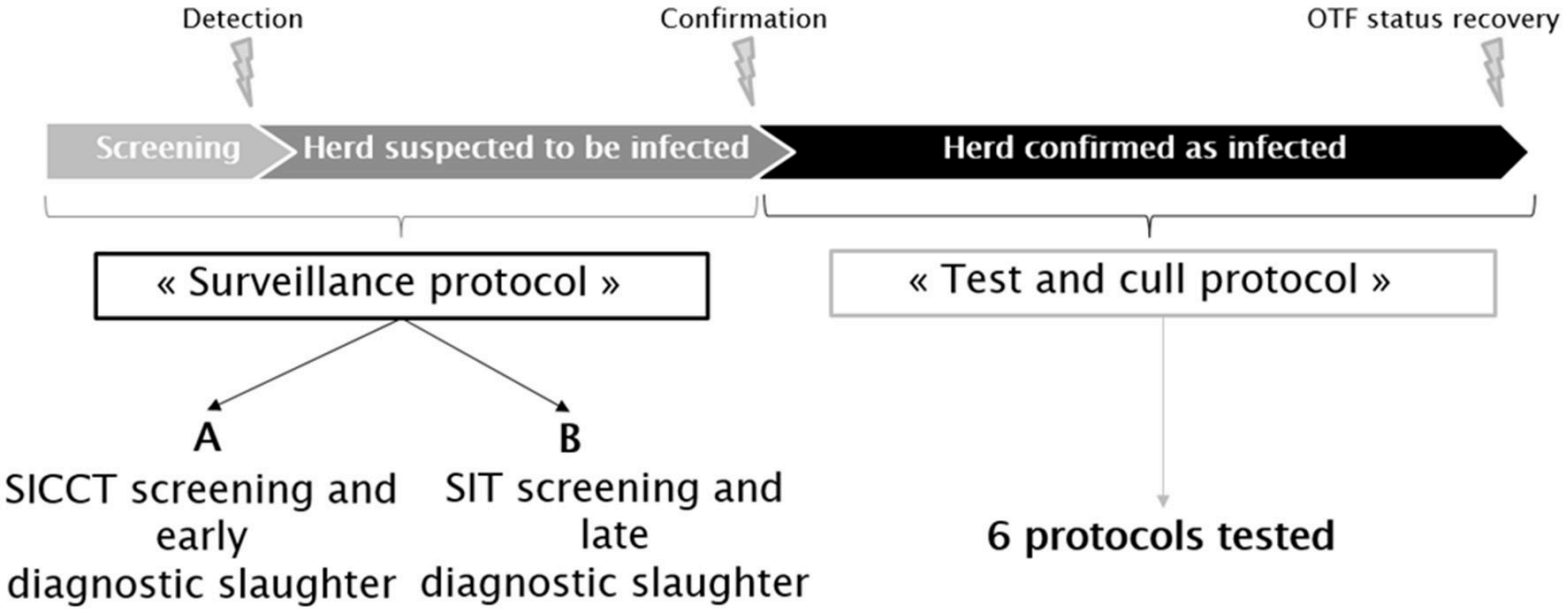
Schematic representation of the bTB surveillance and control protocols applied in France in accordance with Council Directive 64/432/EEC.

Two sets of possible surveillance measures (feasible under present French field conditions) were represented in the model. Herds were assumed subjected to routine screening, based on skin tests of animals over 1 year of age. If at least one reactor was detected, the herd was placed under movement restriction until confirmation (or not) of bTB infection. This phase (from the routine skin testing until the confirmation of infection) will be hereafter called the “surveillance protocol” (Figure 1). Two such surveillance protocols were distinguished. The first one, termed below “A,” used a SICCT annual screening of the herds, followed by the slaughter of non-negative animals to infirm or confirm the suspicion. The second one, termed below “B,” used a SIT annual screening of the herd. Reactors are retested in the following days with IFN-γ; the non-negative animals being then slaughtered for confirmatory purposes. When bTB infection is confirmed, the herd is kept under movement restriction during the implementation of the test-and-cull protocol, which ends when the herd is reported as free from bTB infection (the OTF status of the herd is recovered).

**Figure 2:**
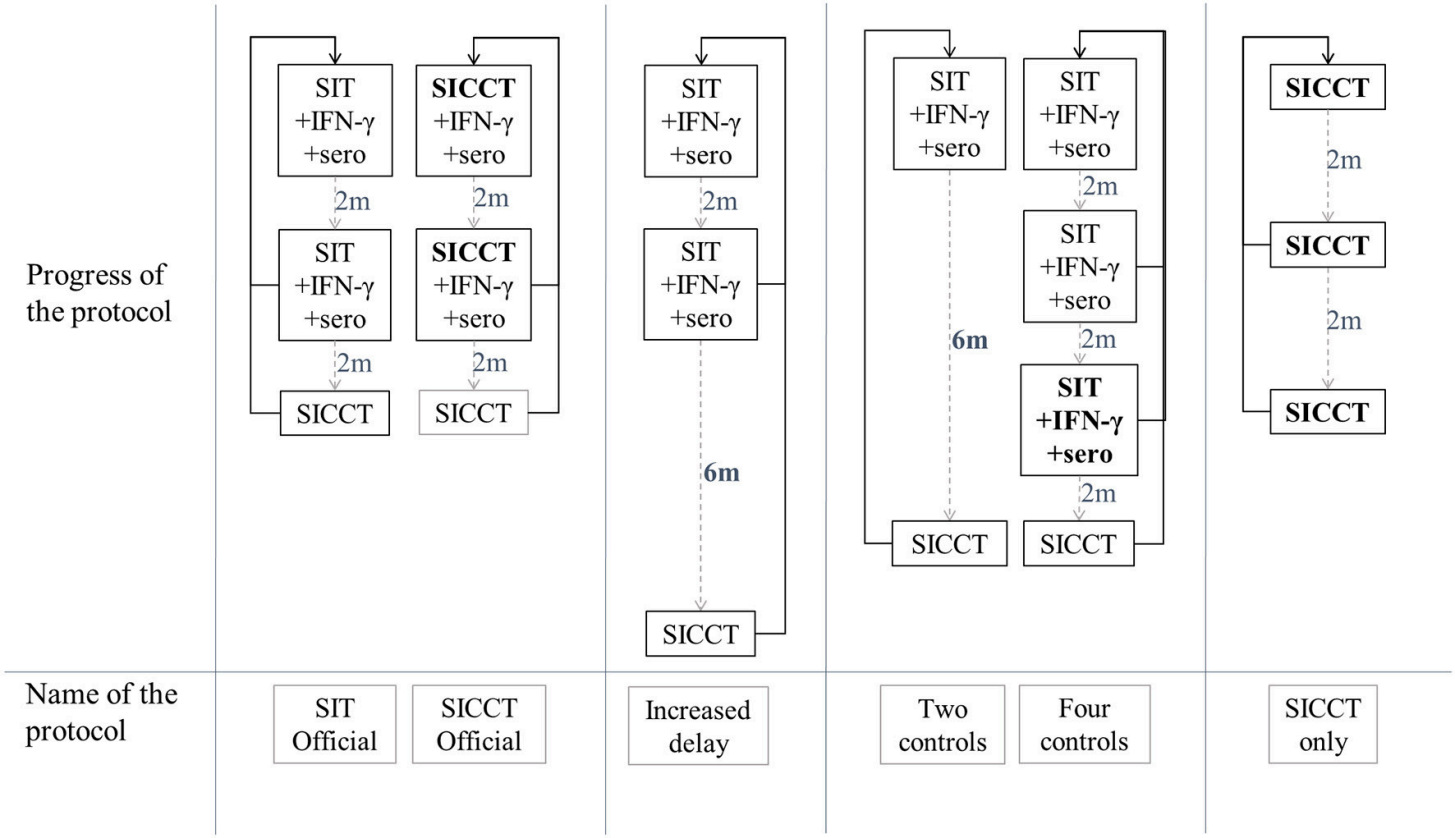
Definition of the studied test-and-cull protocols for bTB control. SIT, single intradermal tuberculin skin tests; SICCT, single intradermal comparative cervical skin test; IFN-γ, gamma interferon test; sero, serology test; 2 m, time period of 2 months; 6 m, time period of 6 months, plain black arrows, non-negative control; dashed gray arrows, negative control.

The current official test-and-cull protocol is based on three series of tests called “controls” (Figure 2) carried out on the entire herd (animals over 1 year of age) every 2 months. The first two controls combine a SIT (“SIT Official” protocol) or a SICCT (“SICCT Official” protocol, in the context of a surveillance of type A), an IFN-γ test, and a serological test. The third control consists of a SICCT. Moving from control X to control X+1 requires all the tested animals to be negative to all tests; otherwise the reactors are culled and the protocol moves back to the 1st control, 2 months later. The OTF status of the herd is recovered when the three controls are negative consecutively.

Based on these two reference protocols, several parameters were modified to specify alternative protocols: the inter-control time periods, the number of controls required and the type of tests used. Four new protocols were thus studied (Figure 2): a protocol where the time period between control 2 and control 3 was increased from 2 to 6 months (called “Increased delay” protocol), a protocol where a control was removed (“Two controls” protocol), a protocol where a control with SICCT, IFN-γ and serology was added (“Four protocols” protocol), and a protocol using only SICCT (“SICCT only” protocol). These scenarios were compared to each other, and to whole depopulation protocol.

Tests sensitivities and specificities were fixed according to the literature and to the expertise of the French national reference laboratory (NRL) for bTB (Table 2).

**Table 2:** Sensitivities and specificities of tests used for bTB surveillance and control in the model of within-herd bTB dynamics.

**Test**	**Sensitivity (median [CI 95%])**	**Specificity (median [CI 95%])**	**References**
SIT	0.81 [0.53; 0.94]	0.91 [0.63; 1.00]	(17)
SICCT	0.75 [0.61; 0.86]	1 [0.99; 1.00]	(17)
IFN bovine and avian PPD	0.70 [0.55; 0.92]	0.94 [0.88; 0.97]	(17)
IFN ESAT6	0.79 [0.64; 0.89]	0.99 [0.98; 1.00]	(17)
Serology	0.60 [0.31; 0.86]	0.93 [0.84; 0.97]	(17)
PCR	0.86 [0.65; 0.96]	1 [1.00; 1.00]	(17)
PCR NRL	1^*^	1	NRL
Histology	0.66 [0.41; 0.84]	1 [0.95; 1.00]	(17)
Culture	0.74 [0.46; 0.94]	1 [0.73; 1.00]	(17)
Routine necropsy (meat inspection)	0.71 [0.37; 0.92]	1 [0.99; 1.00]	(17)
Detailed necropsy (suspected animals)	0.96 [0.82; 1.00]	1 [0.99; 1.00]	(17)

* *In the context of confirmation of positive PCR results obtained by local veterinary laboratories*.

### Indicators

#### Epidemiological effectiveness

The failure probability of the protocol was the probability that a herd would wrongly regain its OTF status while still infected (some infected animals, either latent E or infectious I, are still present but remain undetected). This corresponds to a failure of the system of detection of infected animals (e.g., lack of test sensitivity, animals not tested), and thus of the epidemiological effectiveness of the protocol. The number of infectious animals-months was considered a proxy for the risk of transmission of the infection to the neighboring farms and to the breeder or the farm staff. The number of infectious animals-months was computed as the sum across all infected animals of their total infectivity time (e.g., duration in months spent in I state).

#### Public costs

The proportion of susceptible (S) animals among those culled during the test-and-cull protocol was an indicator of both cost and epidemiological effectiveness, as these false-positive animals are unnecessarily culled and compensated. It was computed as the number of susceptible animals culled during the test-and-cull protocol over the number of culled animals during the test-and-cull protocol (i.e., the number of animals to be compensated).

The overall public costs combined compensations paid to the farmer (for slaughtered animals, calculated based on expert opinions, data provided by French veterinary services (French Ministry of agriculture) of departments Dordogne and Cote d'Or and presented in Table 3) and the laboratory and veterinary costs (prices of analyses, veterinary visits and acts, presented in Table 4). The total farmer compensation costs were calculated for each simulation by summing the average compensation costs of each slaughtered animal, according to its age group. The total laboratory and veterinary costs were calculated by multiplying the number of tests and veterinary visits by their respective unitary costs.

**Table 3:** Average compensation paid to beef cattle farmers per slaughtered animal according to the age group, calculated from compensation reports of seven French herds having been subjected to a test-and-cull protocol between 2014 and 2017, for bTB control.

**Age group**	**Average compensation cost (€)**
0–1 year	507
1–2 years	712
2–3 years	1232
3–6 years	906
6–10 years	758
10–15 years	759

*(Source: French Ministry of Agriculture)*.

**Table 4:** Unit costs of veterinary acts and laboratory analyses used for bTB control in France.

**Act/Analysis**	**Average price (€)**	**Source**
Veterinary visit	27.7	(18)
Blood sample	2.77	(18)
SIT	2.77	(18)
SICCT	6.93	(18)
IFN	50	LDA 24, personal communication, 2017
Serology	12	LDA 24, personal communication, 2017; LDA 21, personal communication, 2017
PCR	50	LDA 24, personal communication, 2017
Culture	50	LDA 24, personal communication, 2017
Histology	50	Pricing grid LAPVSO, Vet diagnostics

#### Acceptability

**Figure 3:**
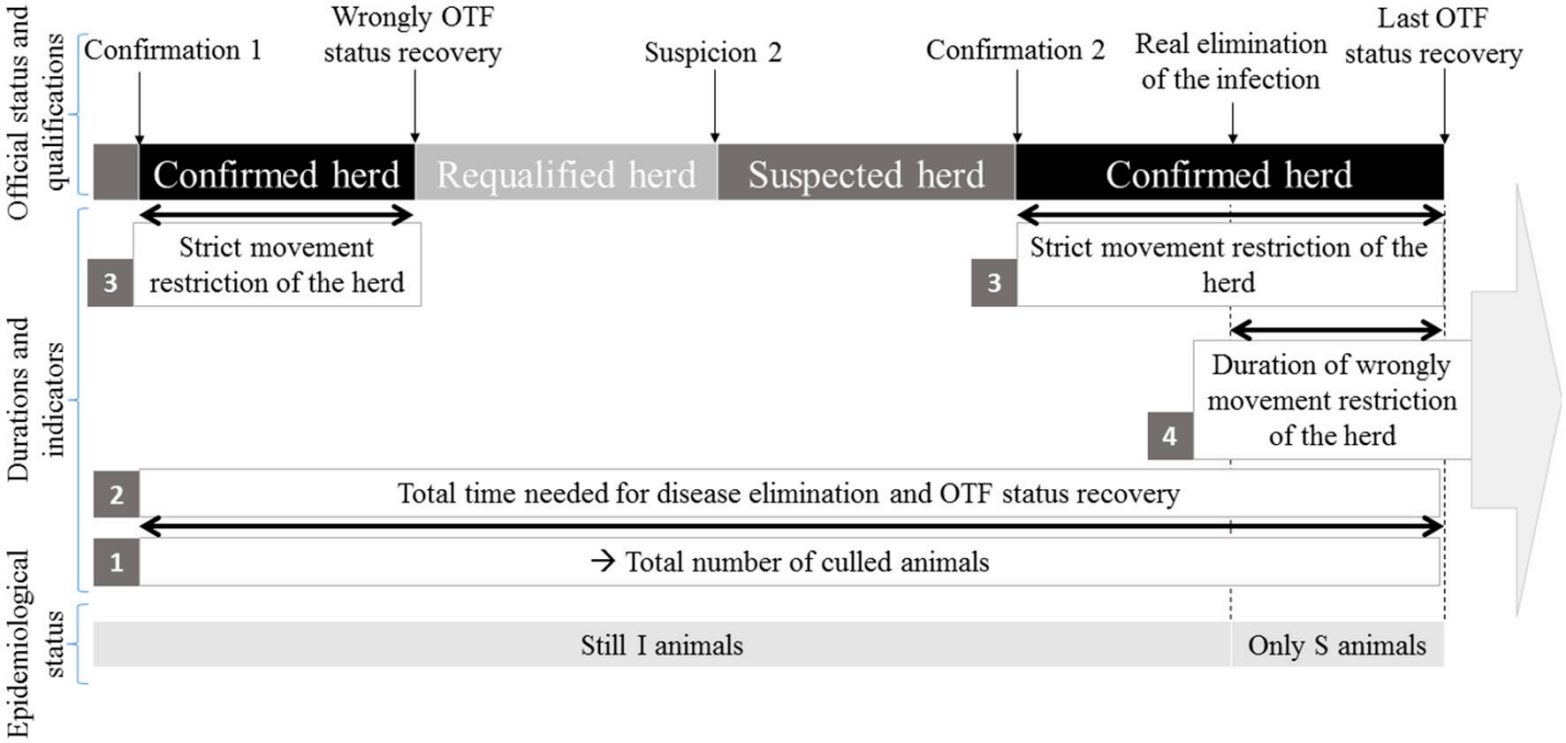
Representation of four indicators [total number of culled animals (1), total time needed for disease elimination and OTF status recovery (2), strict movement restriction of the herd (3), duration of wrongly movement restriction of the herd (4)] in parallel with the evolution of the official and epidemiological status of the herd.

Seven acceptability indicators were defined. First, the total number of culled animals (during surveillance protocol and test-and-cull protocol) was calculated (**Table 7A**, indicator 1 on Figure 3). Besides, the number of culled animal confirmed infected with *M. bovis* by culture (**Table 7A**) was computed as a proxy for acceptability: indeed, the animals culled but not confirmed infected by culture (even if they are infected) are most often seen by breeders as animals “slaughtered for nothing,” which is one of their biggest sources of frustration. The total time needed for disease elimination and OTF status recovery (**Table 7B**, indicator 2 on Figure 3) represented the total time needed for the herd to actually get rid of the infection and correctly regain its OTF status. It could include periods when the herd had temporarily recovered its OTF status but was still infected (see “requalified herd” period while still I animals on Figure 3). The delay between a confirmation and an OTF status recovery represented the duration of strict movement restriction of the herd (**Table 7B**, indicator 3 on Figure 3), which is source of non-acceptability. Modeling allowed computing the duration of the wrongly movement restriction of the herd (**Table 7B**, indicator 4 on Figure 3) by measuring the delay between the slaughter of the last infected animal (E or I) (i.e., the “real elimination of the infection” moment on Figure 3) and the OTF status recovery (Figure 3). The number of veterinary visits (**Table 7A**) corresponded to the number of controls needed to eliminate the infection and recover the OTF status. This number of visits could be greater than the number of controls provided for by the protocol, when one or more controls had been unfavorable, leading to restart the protocol.

The global acceptability was finally evaluated by a synthetic indicator corresponding to the percentage of simulations for which the farmer would have dropped out the test-and-cull protocol before its end (percentage of drop out). The acceptability of the measures by the breeders is a difficult notion to evaluate without sociological survey. It was assumed, according to personal communications from local representatives of veterinary services, that farmers would drop out the protocol if the number of animals confirmed infected exceeded 3, or if the duration of the protocol exceeded 26 months.

For each scenario, 1,000 simulations were conducted, leading to proportions (for binary indicators) or to distributions of indicator values, reported below based on medians, 2.5 and 97.5% percentiles. Finally, three key indicators were represented graphically: the failure probability, the overall public costs, and the percentage of dropout. The six studied test-and-cull protocols were then separately ranked according to each of these three key indicators, and the global rank of each protocol was computed as the average value of these three ranks.

## Results

### Epidemiological effectiveness

Among the test-and-cull protocols (Figure 2), the “Increased delay” had the lowest failure probability: respectively 1.4 and 1.5% after an A (i.e., SICCT annual screening and early diagnostic slaughter) and a B (i.e., SIT annual screening and retesting with IFN-γ before diagnostic slaughter) surveillance protocols (Table 5). On the contrary, the official protocols presented the highest failure probability: 8.3% after an A surveillance protocol (“SICCT Official” protocol) and 12.4% after a B surveillance protocol (“SIT Official” protocol). In general, the failure probability was slightly higher after a B surveillance protocol. After an A surveillance protocol, the number of infectious animals-months remained low regardless of the test-and-cull protocol (median of 1 to 2 animal-months). This number was higher after a B surveillance protocol (median of 3 to 4 animal-months).

**Table 5:** Epidemiological effectiveness indicators of test-and-cull protocols to control bTB in an average French beef cattle herd, compared to whole herd depopulation.

**Protocol**	**Failure probability (%)**		**Number of infectious animals-months**^**a**^	
**Surveillance**	**A**	**B**	**A**	**B**
**Test-and-cull**
SIT Official	7.3	12.4	2 [0–92]	4 [0–140]
SICCT Official	8.3	/^b^	2 [0–80]	/
Increased delay	1.4	1.5	1 [0–50]	4 [0–92]
Four controls	5.0	5.0	2 [0–52]	3 [0–77]
Two controls	4.4	6.5	1 [0–54]	3 [0–111]
SICCT only	7.5	5.4	2 [0–65]	3 [0–89]
**Whole depopulation**	0 by def.	0 by def.	0 by def.	0 by def.

a *Median, brackets: 2.5 and 97.5% percentiles*.

b *“SICCT Official” can only be implemented in the context of a surveillance of type A, which uses SICCT for annual screening*.

### Public costs

Although the difference was moderate, the B surveillance protocol always induced higher public costs than the A one (Table 6B). Whole herd depopulation was clearly the most expensive protocol with a median overall cost of 120 K€ per infected herd. The “Four controls” protocol was the most expensive of the test-and-cull protocols, with median values between 73.2 and 78 K€. However, the others were not much cheaper except the “Two controls” which costed more than 20 K€ less, and the “SICCT only” which was the cheapest protocol, costing between 2.7 and 4.8 K€. In this latter case, very few animals were culled during the protocol (between 0 and 1 in median Table 6A) leading to lower compensations. In addition, this protocol did not use IFN-γ at all, which is an expensive screening test.

Table 6Public costs indicators of test-and-cull protocols to control bTB in an average French beef cattle herd, compared to whole herd depopulation.

**Table:** 

**Protocol**	**Number of culled animals during protocol**^**a**^		**Proportion of S among animals culled during protocol (%)**^**a**^	
**Surveillance**	**A**	**B**	**A**	**B**
**A**				
**Test-and-cull**				
SIT Official	39 [25–107]	49 [28–131]	100 [83–100]	95.9 [66–100]
SICCT Official	20 [10–66]	/	100 [69–100]	/
Increased delay	40 [26–101]	49 [27–135]	100 [83–100]	95.9 [69–100]
Four controls	50 [37–127]	54 [34–131]	100 [88–100]	98.1 [82–100]
Two controls	22 [11–74]	29 [12–90]	100 [79–100]	96.4 [70–100]
SICCT only	0 [0–12]	1 [0–25]	0,0 [0–0]	0 [0–0]
**Whole depopulation**	141	141	100 [94–100]	99.3 [84–100]

**Table:** 

**Protocol**	**Lab/vet costs (K**€**)**^a^		**Compensation costs (K**€**)**^a^		**Overall cost (K**€**)**^a^	
**Surveillance**	**A**	**B**	**A**	**B**	**A**	**B**
**B**						
**Test-and-cull**						
SIT Official	20.1 [18–55]	26.9 [18–69]	35.6 [23–102]	44.8 [25–130]	56 [41–158]	71.7 [43–201]
SICCT Official	20.3 [18–63]	/	18.4 [9–62]	/	40 [27–131]	/
Increased delay	20.2 [18–57]	27.1 [18–69]	36.0 [24–95]	44.2 [25–112]	56.9 [42–151]	71.3 [31–185]
Four controls	27.1 [23–68]	29.5 [22–69]	46.0 [34–115]	48.5 [31–120]	73.2 [58–182]	78 [54–187]
Two controls	11.2 [9–41]	17.7 [9–47]	20.3 [10–68]	26.4 [10–82]	31.6 [20–106]	44.8 [20–126]
SICCT only	2.7 [2–10]	3.6 [2–13]	0.0 [0–12]	0.9 [0–22]	2.7 [2–21]	4.8 [2–35]
**Whole depopulation**	0 [0–1]	0.2 [0–1.8]	120 [115–125]	120 [115–125]	120 [115–125]	120 [116–126]

a *Median, brackets: 2.5 and 97.5% percentiles*.

The proportion of S animals among the animals culled during the protocol was high and reached 100% in median after an A surveillance, for all the protocols except the “SICCT only,” meaning that all infected animals had been culled during the surveillance phase (Table 6A). Oppositely, for the “SICCT only,” this percentage was null, due to the 100% specificity of SICCT. In the other protocols however, almost all culled animals were false positives. After a B surveillance, the percentage was smaller but remained very high (between 95.9 and 99.3% of culled animals in median were S).

### Acceptability

After both A and B surveillances, the “Four control” protocol was the test-and-cull protocol leading to the highest number of culled animals (between 55 and 66 in median) (Table 7A). The “SICCT only” protocol only induced the culling of 6 and 14 animals in median. Whatever the protocol, an A surveillance induced fewer culling (between 13 and 8 animals). After an A surveillance, medians of the number of culture-confirmed animals were null regardless the test-and-cull protocol (Table 7A). Indeed, almost all animals culled were S (Table 6A). The median reached one confirmed animal after a B surveillance, regardless the test-and-cull protocol.

Table 7Acceptability indicators of test-and-cull protocols to control bTB in an average French beef cattle herd, compared to whole herd depopulation.

**Table:** 

**Protocol**	**Total number of culled animals**^**a**^		**Number of culled animal confirmed infected with** ***M. bovis*** **by culture**^**a**^		**Number of veterinary visits**^**a**^	
**Surveillance**	**A**	**B**	**A**	**B**	**A**	**B**
**A**						
**Test-and-cull**						
SIT Official	39 [25–119]	49 [28–138]	0 [0–6]	1 [0–20]	3 [3–9]	4 [3–10]
SICCT Official	20 [10–74]	/	0 [0–7]	/	3 [3–9]	/
Increased delay	40 [26–111]	49 [27–135]	0 [0–6]	1 [0–22]	3 [3–8]	4 [3–10]
Four controls	55 [42–138]	66 [47–159]	0 [0–6]	1 [0–12]	4 [4–10]	5 [4–11]
Two controls	27 [16–89]	40 [22–123]	0 [0–6]	1 [0–15]	2 [2–6]	3 [2–7]
SICCT only	6 [3–29]	14 [4–49]	0 [0–6]	1 [0–17]	3 [3–10]	4 [3–11]
**Whole depopulation**	141 (by definition)	141 (by definition)	0 [0–4]	1 [0–9]	/	/

**Table:** 

**Protocol**	**Total time needed for disease elimination and OTF status recovery (months)**^a^		**Duration of strict movement restriction (months**)^a^		**Duration of wrongly movement restriction (months)**^a^		**Percentage of drop out (%)**	
**Surveillance**	**A**	**B**	**A**	**B**	**A**	**B**	**A**	**B**
**B**								
**Test-and-cull**								
SIT Official	6 [6–32]	8 [6–34]	6 [6–11]	8 [6–17]	6 [4–7]	6 [4–7]	10.7	22
SICCT Official	7 [6–32]	/	7 [6–11]	/	6 [5–7]	/	11.3	/
Increased delay	10 [10–26]	12 [10–28]	10 [10–25]	12 [10–27]	10 [8–11]	10 [8–11]	8.5	20.1
Four controls	8 [8–33]	10 [8–34]	8 [8–20]	10 [8–21]	8 [6–9]	8 [6–9]	9.6	16
Two controls	8 [8–33]	10 [8–34]	8 [8–20]	10 [8–21]	8 [6–9]	8 [6–9]	7.8	21.1
SICCT only	7 [6–33]	8 [6–34]	6 [6–18]	8 [6–21]	6 [6–6]	6 [6–7]	12.8	22

a *Median, brackets: 2.5 and 97.5% percentiles*.

The median number of veterinary visits (or control sessions) needed to eliminate the infection and recover the OTF status showed that in most cases, after an A surveillance, the minimal number of controls was performed (i.e., the number of controls provided for by the protocol), although in some cases, the number of required visits was important (97.5% percentile between 6 and 10 visits). After a B surveillance however, an additional control, in median, was necessary to eliminate the infection and recover the OTF status (Table 7A). After an A surveillance, the total time needed for disease elimination and OTF status recovery varied in median between 6 months for the “SIT Official” protocol and 10 months for the “Increased delay” one (Table 7B). After a B surveillance, this duration was always about two months longer in median, varying from 8 months for the “SIT Official” and “SICCT only” protocols to 12 months for the “Increased delay” protocol. In whole depopulation, the regulatory depopulation duration is 30 days. However, it is necessary to add the time needed for the cleaning-disinfection operations, the repopulation, and the realization of the tests on the new animals to recover the OTF status. This duration is therefore hardly comparable to that of test-and-cull protocols.

Regardless the surveillance protocol, the median duration of strict movement restriction was similar to the total time needed for disease elimination and OTF status recovery (Table 7B). This shows that in most cases, there were no periods of herd infection with false OTF status. However, the 97.5% percentiles showed that these two durations could differ up to 21 months. Thus, these situations were rare, but when they occurred, they lasted a long time (between 6 and 21 months), except for the “Increased delay” protocol where the difference was only 1 month. Duration of the wrongly movement restriction was also most of the time equal to the total time needed for disease elimination and OTF status recovery after an A surveillance (Table 7B). This means the infection was eliminated from the herd when the last infected animals were culled, during the surveillance period. The necropsy confirming the infection, the herd was declared infected and the test-and-cull protocol started although there was no more infected animal. Only the “SICCT Official” and “SICCT only” scenarios had differences of 1 month. However, after a B surveillance, the median durations of wrongly movement restriction were shorter than the total time needed for disease elimination and OTF status recovery. Infection was eliminated from the farm 2 months in median after the confirmation and the implementation of the test-and-cull protocol.

After an A surveillance, the “Two controls” protocol had the lowest percentage of drop out: 7.8% (Table 7B). After a B surveillance however, the “Four control” protocol was the one with the lowest but still quite high percentage of drop out of 16%. The “Two controls” implied 21.1% of drop out. “SICCT only” had the higher percentage of drop out in both A and B cases, but implied 12.8% of drop out after an A surveillance while it reached 22% of drop out after a B surveillance, like the “SIT Official” protocol.

Indeed, considering the percentage of drop out as the main indicator for acceptability, we can note that acceptability was better after an A surveillance, regardless the test-and-cull protocols.

### Global analysis and ranking of the protocols

**Figure 4:**
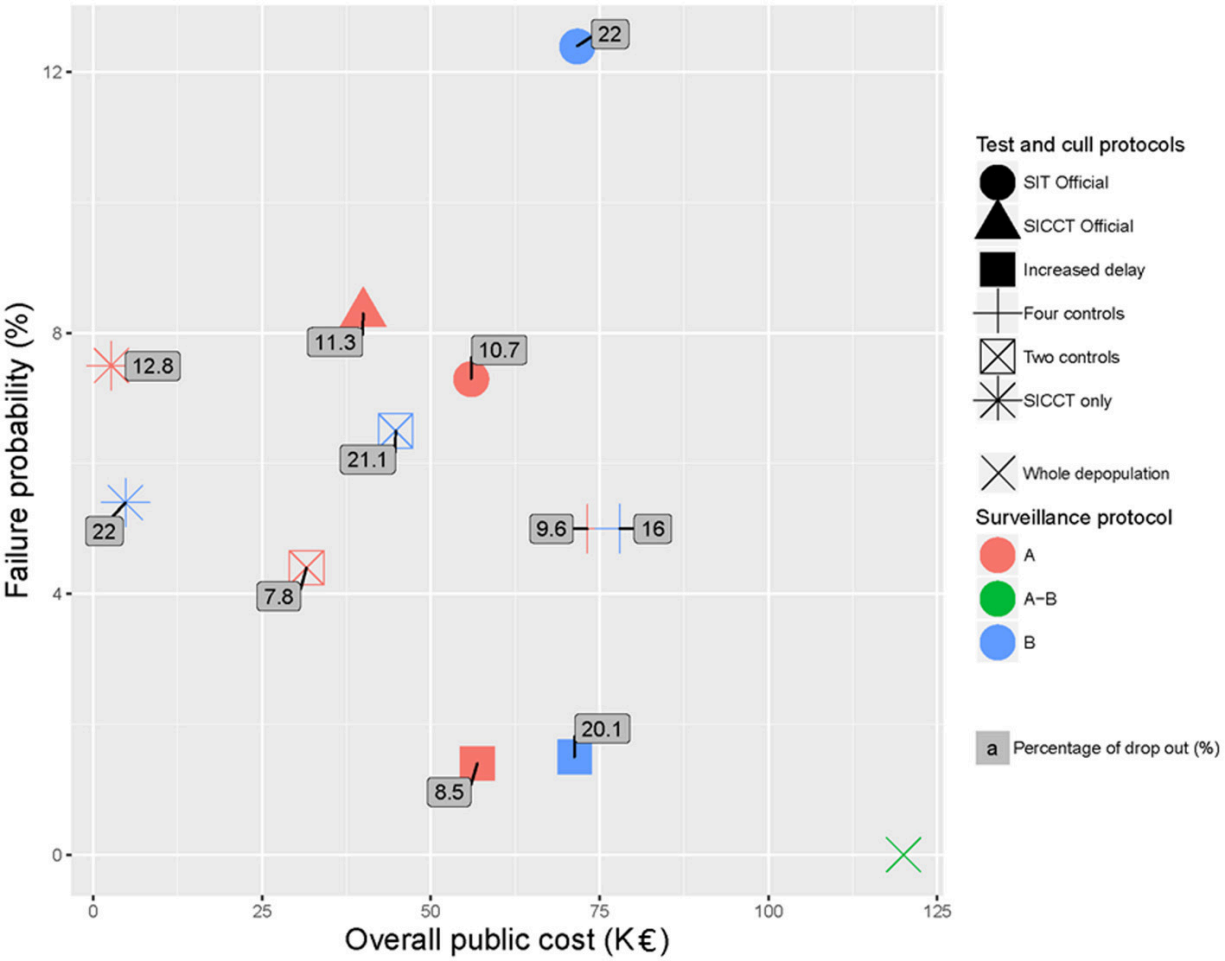
Failure probability of the test-and-cull protocols for bTB control after both A and B surveillances schemes, according to their overall public cost and their percentage of drop out (labels) compared to whole depopulation.

The failure probability of the protocols function on their overall public cost is plotted in Figure 4. The percentage of drop out is indicated as a label for each protocol. In this representation, the optimal protocols should be located in the lowest left part of the graph, (attention needs also to be paid to the acceptability). Figure 4 confirms that B surveillance (blue markers) induced increased public costs (blue markers being always on the right of red markers) and decreased acceptability (superscripts of blue markers being always higher than superscripts of red ones) and effectiveness (blue markers being higher than red ones except for the “SICCT only” protocol). These considerations should lead to prefer the A surveillance protocol. Each scenario was then ranked for each indicator separately (Table 8). Figure 4 and Table 8 show that in both A and B cases, the “SICCT only” protocol was very economical. However, after an A surveillance its failure probability was the second highest (ranked 6th out of 7 protocols, see Table 8) and its percentage of drop out was the highest (ranked 7th out of 7). After a B surveillance, although its failure probability was enhanced (ranked 4th out of 6), its percentage of drop out was the second highest (ranked 4th out of 6, placed equal with “SIT Official”). Therefore, in both cases, the “SICCT only” protocol does not appear to be appropriate for bTB control. The “SIT Official” protocol was among the less effective and most expensive ones, especially after a B surveillance. Its percentage of drop out was the highest after a B surveillance (placed equal with the “SICCT only”) and the 4th highest after an A surveillance. Similarly, the “SICCT Official” protocol was poorly ranked for each of the three indicators.

**Table 8:** Ranking of test-and-cull protocols to control bTB in an average French beef cattle herd, compared to whole herd depopulation, according to three key indicators, and average rank considering the three indicators being at the same level of importance.

**Protocol**	Rank for failure probability^*^		Rank for overall public cost^*^		Rank for percentage of drop out^*^		Mean rank^**^	
**Surveillance**	**A**	**B**	**A**	**B**	**A**	**B**	**A**	**B**
**Test-and-cull**								
SIT Official	5	6	4	4	4	4	4.3	4.7
SICCT Official	7	/	3	/	5	/	5	/
Increased delay	2	2	5	3	2	2	4	2.3
Four controls	4	3	6	5	3	1	4.3	3
Two controls	3	5	2	2	1	3	2	3.3
SICCT only	6	4	1	1	6	4	4.3	3
Whole depopulation	1	1	7	6	7	6	5	4.3
Total number of protocols	7	6	7	6	7	6	7	6

* *1is the protocol with the lowest value, 6 or 7 is the one with the highest one*.

** *The smaller the rank, the better the scenario*.

After an A surveillance (red markers), the “Two controls” and “Increased delay” protocols appeared to be the two best protocols. Both had a reasonable acceptability (<9% of drop out, 1st and 2nd rank), a reasonable failure probability (<5% of failure, 2nd and 3rd rank) and induced reasonable public costs (<60 K€TF and 2nd and 5th rank). The “Two controls” protocol was cheaper but had a higher failure probability whereas the “Increased delay” one was almost twice more expensive but with a much lower failure probability.

The average of the three ranks (failure probability, public costs and drop out percentage) allowed obtaining a global ranking of these protocols, while attributing the same importance to each of the three criteria (Table 8). The “Two controls” protocol was the best tradeoff (average rank of 2) after an A surveillance. After a B surveillance, the best tradeoff was obtained by the “Increased delay” protocol with an average rank of 2.3, closely followed by the “Four controls” and “SICCT only” protocols (average rank of 3) (Table 8).

## Discussion

In this study, we evaluated the official test-and-cull protocols implemented in France, in accordance with Council Directive 64/432/EEC as well as four alternative protocols. Their epidemiological effectiveness, the public costs they induce and their acceptability to farmers were compared.

For that purpose, a previously published and validated model was used (12), model that allowed representing the “true” health state of the animals and the detection and control events, in order to quantify events such as incorrectly assigning a bTB free status to the farm (19, 20). This model was chosen because it allowed parameterizing easily alternative surveillance and control protocols, and because its parameters were estimated from field data collected in France, and validated independently: Bekara et al. (12) performed both an internal validation using a leave-one-out cross-validation procedure, and an external validation to demonstrate the ability of the model to reproduce observational bTB data collected in France between 1980 and 2010, that were not used for parameter estimation. The model was parameterized based on French data with an average beef herd size (141 animals) that may be greater than in most of European countries. According to the Directorate General of the European Commission responsible for statistical information at Community level (Eurostat), the last calculated average size of beef herds would be 70 animals (21). The model could easily be adapted to different breeding contexts.

Results suggest that the official protocol could probably be improved, as alternative protocols appeared more effective, acceptable while inducing lower public costs.

The proportion of non-infected animals among the slaughtered animals appeared high, except with the “SICCT only” protocol. Following an A surveillance (based on SICCT annual screening and slaughter of positive animals to confirm suspicions), *M. bovis* was often very quickly eliminated from the infected farms (i.e., either at the time of the confirmation of infection, or in the first months following this confirmation). Moreover, the low specificity of the SIT and IFN-γ tests (17) led to the cull of many susceptible animals. This phenomenon was mitigated when using surveillance protocol B (based on SIT annual screening, IFN-γ on positive animals and slaughter of non-negative animals to confirm suspicions), but the simulated proportion of non-infected animals among the culled animals remained high, with a minimum value of 95.9% for protocols other than the “SICCT only.” Indeed, even though the simulated scenario rankings were relatively similar following an A or a B surveillance, the surveillance protocol had a strong impact on the epidemiological effectiveness, the public costs and the acceptability to farmers. After an A surveillance, and therefore a drastic management of the suspicion, most herds did not contain infected animals at the start of the test-and-cull protocol, unlike after a B surveillance. Epidemiological effectiveness, costs and acceptability of the test-and-cull protocols were then enhanced. For example and according to acceptability, herds managed under surveillance protocol A had a shorter duration of strict movement restriction (about 2 months in median) than those managed under surveillance protocol B. The farmer can therefore be prepared to the fact that the protocol will last longer if the surveillance protocol of his herd was B.

We investigated the balance between the costs that can be invested in test-and-cull protocols and the consequences of choosing a specific scheme. It all depends on the goal: if it is to eradicate the infection, the most effective and acceptable protocols will have to be chosen, regardless of their cost. Indeed, without good acceptability, the actors will not follow the measures and the strategy will lose of power. In this case, “Increased delay” protocol is to be implemented. If, however, one is willing to accept a small percentage of outbreaks wrongly regaining their bTB free-status, then less effective but less expensive scenarios may be chosen, such as the “Two controls” protocol in the context of reducing public expenditure. In both cases, the good effectiveness of the “Increases delay” and “Two controls” protocols highlights the importance of long inter-control delays for the epidemiological effectiveness of the scenarios. This appears to be valid even when very few animals are actually infected.

Field observations bring support to the results we obtained. According to a study on the typology of French farms that were subjected to the test-and-cull official protocol between 2014 and 2017 (22), infection was laboratory-confirmed in <4 animals in 95% of the farms. In our simulations, we obtained a very low number of laboratory-confirmed infected animals (between 0 and 1 in median) for the official scenarios, which is thus consistent with field observations. In the same way, the number of control sessions observed in the field (on average 3.3 visits) was close to the figure we obtained (median of 3 to 4 visits for the official protocols).

In the model, births were assumed to compensate for animal culls, herd size thus remaining constant. This simplifying assumption may not accurately represent reality. First, some breeders decide to reduce the size of their herd when starting the test-and-cull protocol, for reasons of biosecurity and easier management of the batches (personal communication 2017: F. Chevalier, French national referent for bTB). Then, if many animals are slaughtered (rightly or wrongly), it can surpass the amount of births and prevent renewal. Thus, the number of young cattle in the model could be over-estimated. However, these young cattle probably play a minor role in infectious dynamics and are, in practice, not tested because they are too young. The impact of this simplification on our results is therefore assumed to be low.

The overall indicator of acceptability (percentage of drop out) was calculated taking into account the number of confirmed animals and the duration of the test-and-cull protocol. The number of animal reacting to tests may also have been a relevant parameter, but it was too difficult to determine an adequate threshold. In the field, when the number of reactors is high, veterinary services can advise or even force the breeder to shift from the test-and-cull protocol to whole herd depopulation. However, no official or empirical value exists to support that. This is why we decided not to take into account this parameter for the definition of acceptability.

The six studied test-and-cull protocols have been compared and ranked according to 3 key indicators, and according to an overall rank that gave the same level of importance to each. However, in real life, the choice of a control strategy does not always obey a pure epidemiological, economic or social rationality (23). Using a multicriteria decision analysis method (MCDA) would allow investigating more precisely the overall ranking of the protocols according to the expectations of the decision-makers (24).

Even though we focused on test-and-cull protocols applied or feasible in France, some of the protocols we analyzed are also relevant at the European level. The bTB surveillance and control protocols used in EU countries are indeed not country-specific, as these protocols and the tests they include stem from the European legislation (especially the Council Directive 64/432/ECC). As an example, test and cull is very common in the United Kingdom, where bTB can reach in some areas the highest prevalence of the EU (except in Scotland which is OTF), or in Spain (1, 4). For example, England and Northern Ireland eradication program incorporate the surveillance protocol we called “A” (13, 25, 26). A SICCT is performed on the whole herd and reactors are immediately removed for slaughter. If postmortem evidence of *M. bovis* infection cannot be demonstrated in any of the slaughtered reactors, OTF herd status that was suspended may be restored after one single skin test of all the animals with negative results, minimum 60 days later. However, if the infection is confirmed or if more than two animals had reacted to the SICCT, the herd loses its OTF status and enters in a “test and cull protocol” close to one of those we tested: the “SICCT only.” Indeed, two consecutively negative SICCT on the whole herd are required to restore the OTF herd status while we modeled a three control protocol. Although other protocols used in EU countries could easily be implemented and compared using the model we used (which was designed for that purpose), it can be expected that the main results would remain valid, such as the reduced failure probability when inter-control delays are lengthened, or the positive effect of type A surveillance on epidemiological effectiveness, on cost and acceptability.

In conclusion, this study aimed at contributing to the identification of points on which decision-makers should act to improve the detection and control of bTB. It appears that there is room for an improvement of the present official test-and-cull protocol implemented in France in line with Council Directive 64/432/EEC, in particular by allowing an increase in the time interval between controls. Decision-makers may use this study to communicate with field actors and justify the needed modification of the French regulations concerning the bTB control. As tests and protocols are close between European countries it could be interesting to extend this study to other regions facing bTB taking into account their specificities in terms of epidemiological situation and cattle breeding.

## Author contributions

HL, MS, AC, and BD conceived and designed the study. HL, MS, and BD encoded the model and indicators. HL, MS, AC, and BD performed the analysis. HL wrote the manuscript. HL, MS, AC, and BD revised the manuscript. All the authors approved the submitted version of the manuscript.

### Conflict of interest statement

The authors declare that the research was conducted in the absence of any commercial or financial relationships that could be construed as a potential conflict of interest.
